# Effect of free-weight vs. machine-based strength training on maximal strength, hypertrophy and jump performance – a systematic review and meta-analysis

**DOI:** 10.1186/s13102-023-00713-4

**Published:** 2023-08-15

**Authors:** Markus E. Haugen, Fredrik T. Vårvik, Stian Larsen, Arvid S. Haugen, Roland van den Tillaar, Thomas Bjørnsen

**Affiliations:** 1 Department of Sport Sciences and Physical Education, Nord University, Levanger, Norway; 2 Department of Sport Science and Physical Education, Faculty of Health and Sport Sciences, University of Agder, Kristiansand, Norway; 3 Strength and Power consultant at the Norwegian Olympic and Paralympic Committee and Confederation of Sports, Oslo, Norway; 4 Norwegian Olympic and Paralympic Committee and Confederation of Sports, Oslo, Norway; 5 Department of Anaesthesia and Intensive Care, Haukeland University Hospital, Bergen, Norway; 6 Department of Nursing and Health Promotion Acute and Critical Illness, Faculty of Health Sciences, OsloMet – Oslo Metropolitan University, Oslo, Norway

**Keywords:** Resistance training, Equipment, Exercise, Stability, Modalities, Force production, Muscle size

## Abstract

**Background:**

The effectiveness of strength training with free-weight vs. machine equipment is heavily debated. Thus, the purpose of this meta-analysis was to summarize the data on the effect of free-weight versus machine-based strength training on maximal strength, jump height and hypertrophy.

**Methods:**

The review was conducted in accordance with the preferred reporting items for systematic reviews and meta-analyses (PRISMA) guidelines, and the systematic search of literature was conducted up to January 1^st^, 2023. Studies that directly compared free-weight vs. machine-based strength training for a minimum of 6 weeks in adults (18–60 yrs.) were included.

**Results:**

Thirteen studies (outcomes: maximal strength [*n* = 12], jump performance [*n* = 5], muscle hypertrophy [*n* = 5]) with a total sample of 1016 participants (789 men, 219 women) were included. Strength in free-weight tests increased significantly more with free-weight training than with machines (SMD: -0.210, CI: -0.391, -0.029, *p* = 0.023), while strength in machine-based tests tended to increase more with machine training than with free-weights (SMD: 0.291, CI: -0.017, 0.600, *p* = 0.064). However, no differences were found between modalities in direct comparison (free-weight strength vs. machine strength) for dynamic strength (SMD: 0.084, CI: -0.106, 0.273, *p* = 0.387), isometric strength (SMD: -0.079, CI: -0.432, 0.273, *p* = 0.660), countermovement jump (SMD: -0.209, CI: -0.597, 0.179, *p* = 0.290) and hypertrophy (SMD: -0.055, CI: -0.397, 0.287, *p* = 0.751).

**Conclusion:**

No differences were detected in the direct comparison of strength, jump performance and muscle hypertrophy. Current body of evidence indicates that strength changes are specific to the training modality, and the choice between free-weights and machines are down to individual preferences and goals.

**Supplementary Information:**

The online version contains supplementary material available at 10.1186/s13102-023-00713-4.

## Key point

If your goal is to maximize strength adaptations, the choice between free-weight and machines should be based on individual preference. The principle of specificity applies, which states that you should choose the exercise you want to be stronger in. This is important for athletes who are tested in specific exercises.

For muscle hypertrophy one should consider individual preference when choosing between modalities. We speculate that the combination could result in more complete hypertrophy.

**Supplementary Information:**

The online version contains supplementary material available at 10.1186/s13102-023-00713-4.

## Background

It is well established that manipulation of resistance training variables such as volume, intensity, frequency, load, exercise selection, exercise order and rest intervals can influence strength and hypertrophic adaptations [1]. However, the stability requirement from different exercise modalities have received less attention. The choice between free-weights and machines are heavily debated by coaches, athletes, and recreational lifters. The debate is whether one should perform resistance exercises with free-weights, which demands more stability and higher stability requirements, or machines with lower stability requirements which require less stabilization, to maximize long-term strength and hypertrophy [2, 3]. Alternatively, the adaptations are simply governed by the principle of specificity with strength, power and hypertrophy gains specific to the exercises performed, but equal effect size if strength and power is measured neutral tests and hypertrophy across all total muscle mass used [4, 5]. Free-weights often refer to exercises with dumbbells and barbells, whereas machines can be defined as a device that contains a pin-loaded weight stack. In most circumstances “machine-based strength training” refers to exercises that are performed with a fixed plane of motion, thus guiding the resistance through a specific path, which reduces the requirement from synergistic muscles [6]. On the other hand, free free-weight exercises will normally let you dictate the plane of motion more flexibly and thus induces more variation in lever arms [6, 7].

American College of Sports Medicine argue that machines may be safer to use than free-weights based on skill requirements [1]. This is further supported by Kerr [8] who reported a higher injury rate with free-weights, but most of the injuries were related to weights falling on people, and not the execution of the modality [8]. However, Fisher [9] and ACSM [1] mainly refer to injury risk in cross-sectional studies, and only a few longitudinal experimental studies, making it questionable if a causal relationship exists between modalities/stability and injury risk. Summed up, it is uncertain if there are different injury risks between free-weight and machine-based strength training. It could be speculated if the most important considerations in the incorporation of free-weight exercises versus machines is the experience of the person who trains, familiarity with specific exercises, and the primary goal with the training (i.e., powerlifters and Olympic weightlifters must train with free-weights due to the nature of the sport, but bodybuilders and recreational can choose either or both) [1].

Free-weight exercises demand greater coordination between muscles to execute the exercises properly due to higher instability of the movement [7, 10], and the movements are often more similar to daily life activities than machine-based exercises. Therefore, free-weight exercises are often suggested to be more “functional” than machine-based exercises [1] with a better transfer to improvements in daily physical function [11]. Moreover, there is believed often suggested that free-weights have a higher transferability of strength due to the performance requirements on unstable conditions [12]. The greater coordination demand with free-weight exercises, such as squat and bench press can result in a higher myoelectric activity in synergist muscles than machine equivalent exercises [6, 13]. This is further supported by Schwanbeck [14], who observed higher myoelectrical activation in the synergist muscles in the lower limbs when performing barbell back squats compared to smitch machine squats. The increased myoelectrical activity in the synergists could potentially result in more muscle growth across several involved muscles, and thus lead to more total growth. However, the relationship between acute myoelectrical activity and long-term musculoskeletal hypertrophy is uncertain [15, 16], and the higher myoelectrical activity may also be due to greater stability demands.

There has been observed a higher maximum load lifted with machine-based exercises compared to free-weights within a similar exercise or movement. For example, some researchers have shown that more loads can be lifted with a Smith machine squat compared to a free-weight squat [14, 17]. This could be affected by stability and/or bar path. A possible explanation is that it seems that the muscles prioritize stability over force production in the direction of the desired exercise movement [18, 19]. Hence, higher stability requirements negatively affect strength performance. The higher load in the machine-based exercise could possibly affect strength gains beneficially after a period of training through load dependant mechanisms such as mechanical tension and neural adaptations [20], However, it will likely not lead to more muscle growth as varying load ranges seems to affect hypertrophy similarly if training is performed close to failure [21, 22].

To provide greater understanding of the differences between free-weights and machine-based strength training, Heidel and colleagues performed a meta-analysis in which they pooled studies comparing the two modalities [5]. They found that free-weights increased strength the most when they tested free-weights and machines increased the strength the most when tested in machines. Whilst no difference was observed for neutral strength, power, and muscle hypertrophy. However, only three studies assessing hypertrophy was available. Since then, two additional studies measuring hypertrophy have been published [23, 24]. Moreover, additional studies had been published for strength and jump performance. Thus, an updated meta-analysis would have more statistical power. Furthermore, as mentioned by ACSM [1], Lavallee & Balam [25] and Fisher [9], training experience could influence the outcome but is yet to be explored. In addition, comparing the effect size of strength changes in the modality each group trained, such as free-weight training and changes in free-weight strength vs. machine training and changes in machine strength has not been conducted. Therefore, in this review we will provide an updated meta-analysis on the influence of free-weights and machine-based strength training on maximal strength, jump performance and hypertrophy, including sub analyses on training experience and separate upper-body and lower-body strength analysis.

## Methods

This review followed the Preferred Reporting Items for Systematic Reviews and Meta-Analyses guidelines [26]. The review protocol was preregistered in PROSPERO (CRD42021270740).

### Literature search

The literature search was performed in the databases MEDLINE and Embase via Ovid and SPORTDiscus via EBSCOhost. Last search date was set to 1^st^ January 2023. Searches were carried out using a combination of keywords and MeSH terms (described in detail in the appendix), with the following keywords and associated synonyms: (“resistance/strength exercise/training”) and (“free weight*” and machine*) and (strength, power, jump, CMJ, force, fat free mass, lean mass, hypertrophy or “muscle size/thickness”).Secondary searches were performed for: (a) screening reference lists of the included studies; (b) forward and backwards citations; and (c) search through the authors private library. F.T.V performed the searched and exported it to Rayyan (https://www.rayyan.ai/). M.H and S.L performed the study selection blinded for each other. Any disagreement was solved with a discussion with F.T.V and T.B. Titles and abstracts of the initially identified studies were first screened based on predefined inclusion criteria. A full-text review was performed if a decision based on title/abstract was not possible. The included full texts were reviewed by M. H and discussed with S.L and F.T.V. Any disagreement was resolved with discussion until a consensus was reached.

### Inclusion criteria

Articles were considered eligible for this systematic review and meta-analysis if they met the following criteria: (a) experimental design; (b) published in a peer-reviewed, English-language journal; (c) comparing free-weights and machines; (d) had a minimum duration of 6 weeks; (e) only included adults (18 – 60 years old) free from chronic disease or injury; and (f) included at least one method of estimating changes in muscle mass and/or measured maximal strength and/or jump height or power. Other forms of customized machines/equipment such as haptic-based, pneumatic- or variable resistance, and freemotion/cable machines without a fixed-path resistance, were excluded.

### Data extraction

The following data were extracted from the included studies and tabulated on a predefined Microsoft excel coding sheet (Version 16.42): first authors name and year of publication, duration (weeks), participants, gender, training status, exercises trained for the free-weight and machine group, an overview of the training programme and hypertrophy, maximal strength, and countermovement jump height outcomes (pre-post means ± standard deviations). During data extraction we noticed that the data in Saeterbakken [27] and Saeterbakken [28] were similar. We contacted the lead author who confirmed it so only the data from the 2016 study was used to avoid double counting. When necessary, the corresponding author of the study was contacted to request required information.

### Classification of training status

To classify participants as “experienced resistance trained”, we used free-weight strength data from Santos Junior [29] where the threshold was set from their advanced training status estimate. For men to be classified as “trained”, the free-weight group had to lift > 100% of their body mass in bench press, > 120% of their body mass in barbell back squat and > 150% of their body mass in the deadlift. For women to be classified as “trained”, their strength had to be > 60% of their body mass in bench press, > 100% of their body mass in the barbell back squat and > 120% of their body mass in the deadlift [29]. The mean body mass of the free-weight group and the pre-test strength data were used.

### Methodological quality

The 12-point TESTEX scale [30] was employed to assess the methodological quality of the studies. TESTEX scale is divided into two categories. 5 points for study quality and 10 points for reporting [30]. It consists of twelve questions, but items 6 and 8 have three and two questions, respectively. The maximum number of points is 15. Based on the summary scores, we classified studies; poor quality (< 6 points), fair quality (6–8 points), good quality (9–11 points), or excellent quality (12–15 points) [31]. If the studies fulfilled the criteria they received one point per question, and if not a score of 0 was given. It had to be stated clearly in the article if the criteria were fulfilled or not. In case of uncertainty, no points were awarded [30]. Two authors (M.H. and S.L) carried out the quality assessment. They rated the studies independently, blinded for each other’s ratings to reduce possibility for selection biases. After the first rating, the scores were sent to a third author (F.T.V.) who recoded the scores and forwarded them to another author (A.S.H) that was blinded of which co-author had rated the study quality. A.S.H performed Kappa-analysis and percent agreement of the ratings (Table 1). For the studies from Saeterbakken [27] and Augustsson [32], there were a few minor disagreements on the Testex quality assessment of items 1, 4 and 6. To reach 100% consensus on the quality assessment, disagreements were solved through discussion between the two raters. The final rating scores are listed in Table 1. Items with Kappa < 1.00 or < 100% agreement were finally discussed to reach 100% agreement on the TESTEX quality scale (Table 2).

**Table 1:** Assessment of inter-rater reliability and agreement between raters’ blinded judgement of included studies (*n* = 13) using the Testex screening tool

		Measurements of agreement			
		Kappa	SE	*P*-value	Percentage
1	Eligibility criteria included	0.70^d^	0.18	0.005	86
2	Randomization method stated	1.00^e^	0.00	< 0.001	100
3	Allocation concealment	1.00^e^	0.00	< 0.001	100
4	Groups similar at baseline	NA	-	-	92
5	Assessor blinded	Constant	-	-	100
6a	Study withdrawals < 15%	1.00^e^	0.00	< 0.001	100
6b	Adverse events reported	1.00^e^	0.00	< 0.001	100
6c	Session attendance reported	0.63^d^	0.33	0.011	92
7	Intention-to-treat analysis	Constant	-	-	100
8a	Between-group primary analysis	Constant	-	-	100
8b	Between-group secondary analysis	Constant	-	-	100
9	Point measures for all outcomes	Constant	-	-	100
10	Activity monitoring controls	Constant	-	-	100
11	Relative exercise intensity adjusted	Constant	-	-	100
12	Exercise energy expenditure information reported	Constant	-	-	100
Total scores of intra-rater agreement		0.78^d^	0.14	< 0.001	85

*Abbreviations Testex* Tool for the assEssment of Study qualiTy and reporting in Exercise, *SE*Standard Error, *NA* Not applicable due to constant values preventing Kappa analysis, *Constant* 100% agreement on rating, preventing Kappa analysis. Subscript letters denote level of agreement with Kappa analysis: a =  < 0.20: poor, b = [0.20, 0.40): fair, c = [0.40, 0.60): moderate, d = [0.60, 0.80): good, and e = [0.80, 1.00]: very good [33]

**Table 2:** Characteristics of the studies included in the review

Study	N	Training programme	Exercises free weight group	Exercises machine group	Duration (w)	Strength measurement	Hypertrophy measurement	Training status	TESTEX score
**Aerenhouts & D’hondt 2020 ** [23]	24 men (12 FW & 12 machine)	2 sessions a week with 3 sets of 12 reps on every exercise	**Upper-body:** barbell bent over row, standing shoulder press with dumbbells & dumbbell bench press. **Lower-body:** Barbell back squat & barbell deadlift	**Upper-body:** Chest press, seated row & shoulder press **Lower-body:** Leg press & hip extension	10	1 RM	Circumferences	Untrained	9
**Augustsson et al., 1998 ** [32]	21 (11 FW & 10 machine)	2 sessions a week with 3 sets of 10 reps	Barbell back squat	Leg extension & hip abduction	6	3 RM	NR	Trained	10
**Fry et al., 1992 ** [34]	12 men (7 FW & 5 machine)	3 sessions a week with 3 sets of 10 reps	Barbell back squat	Leg extension & leg press	8	10 RM, MVC	NR	Untrained	7
**Langford et al., 2007 ** [12]	29 men (16 FW & 13 machine)	2 sessions a week. 3 sets with 12 reps in week 1, then 5 sets of 4–6 reps	Barbell bench press	Machine chest press	10	3 RM	NR	Trained	7
**Lennon et al., 2010 ** [35]	304 men (173 FW & 131 machine)	3 sessions a week, core lifts 3 sets with 10–12 reps’ week 1–5, 3 sets with 6–8 reps week 6–9 and 3 sets with 3–5 reps week 10–12. Auxiliary lift was performed with 3 sets of 10–12 reps	Free weight bench press	Motion specific machines	12	1 RM	NR	Trained	6
**Mayhew et al., 2010 ** [36]	90 women (38 FW & 52 machine)	3 sessions a week, core lifts were performed with 3 sets of 10–12 reps’ week 1–4 and 3 sets of 6–8 reps week 6–8. Auxiliary lifts were performed with 3 sets of 10–12 reps’ week 1–8	Free weights	Motion specific machines	12	1 RM	Skinfold	Untrained	8
**Prieto-Gonzalez et al., 2021 ** [24]	22 men (11 FW & 11 machine)	2 sessions a week. 3 sets of 12 reps in week 1&2, 3 sets with 12–10-8 reps in week 3&4, 3 sets of 8 reps in 5&6 and 3 sets of 6 reps in week 7&8	**Upper body:** Weighted crunches, dumbbell press, one arm dumbbell row, triceps kickback, alternative dumbbell curl. **Lower body:** Lunges with olympic bar, romanian chair weighted back extension	**Upper-body:** abdominal crunch on machine, machine back extension, chest press, rowing machine, tricep dip machine, bicep curl on scott machine **Lower-body:** Leg press	8	1 RM, CMJ	Skinfold	Untrained	7
**Rossi et al., 2018 ** [37]	17 men (8 FW & 9 machine)	2 sessions a week, 6 sets with 8–12 reps	Barbell back squat	Leg press	10	1 RM, CMJ	Bod pod	Trained	9
**Saeterbakken et al., 2019 ** [4]	24 men (11 FW & 13 machine)	13 sessions in total. Session 1–4 were conducted 3 sets of 10 reps, session 5–10 with 4 sets of 8 reps and session 11 and 12 with 4 sets of 6 reps	Barbell back squat	Smith machine squat	7	10 RM, MVC, CMJ	Ultrasound	Trained	9
**Saeterbakken et al., 2016 ** [27]	25 men (13 FW & 12 machine)	2 sessions a week. 4 sets of 6 reps	Dumbbell bench press	Smith machine bench press	10	6 RM	NR	Trained	9
**Schwanbeck et al., 2020 ** [38]	36 (7 men and 11 women FW & 8 men and 10 women machine)	4 sessions a week, 4 sets on each exercise, 8–10 rep’s week 1–3, 6–8 reps week 4–6 and 3 sets of 4–5 reps week 7 and 8	**Upper body**: Flat barbell bench press, incline barbell bench press, bent over barbell row, chin-ups. supine elbow extension, dumbbell kickback, dumbbell shoulder press, dumbbell lateral raise, camber bar curl & preacher curl **Lower-body:** Barbell back squat, straight leg deadlift, lunge & single-leg calf raises	**Upper-body**: Smith machine bench press, smith machine incline bench press, seated row, lat pulldown, machine triceps press-down, rope press-down, shoulder press, machine lateral raise, machine biceps curl & machine preacher curl. **Lower body:** Smith machine squat, leg extension, seated hamstring curl & machine calf raise	8	1 RM & 6–10 RM	Bod pod & ultrasound	Untrained	7
**Schwarz et al., 2019 ** [39]	18 women (9 FW & 9 machine)	2 sessions a week, 3 sets of 10–12 reps’ week 1, 3 sets of 8–10 reps week 2, 4 sets of 6–8 reps in week 3, 5 sets of 6–8 reps in week 4, 5 sets of 3–5 reps in week 5 and 6 sets of 3–5 reps in week 6	Barbell back squat	Hack squat	6	1 RM, CMJ	NR	Trained	8
**Wirth et al., 2016 ** [40]	83 men (43 FW & 40 machine)	2 sessions a week, 5 sets of 8–10 reps’ week 1–3, 5 sets of 6–8 reps week 4–6 and 5 sets of 4–6 reps week 7 and 8	Barbell back squat	Leg press	8	1 RM, MVC, CMJ	NR	Trained	7

*Note*: *1 RM* 1 repetition maximum, *6 rm* 6 repetition maximum, *NR* not recorded

## Statistical analysis

The meta-analyses of the between-group comparison of free-weight versus machine training groups were the primary analyses. Studies were pooled using the inverse variance method with 95% confidence intervals (CI). Forest plots were generated with random effects modeling to present test statistics as standardized mean differences (SMD) from the continuous values expressed as Hedges’g to adjust for possible small sample bias [41]. Hedges’g SMD and 95% CI were calculated using Comprehensive Meta-Analysis (version 3.3; Biostat Inc., Englewood, NJ) based on sample size, means and pooled pre- and post-SDs for the within-group analyses and pooled post SDs for the between-group analyses. Variation of the true effect size was presented with 95% prediction intervals (PI), calculated based on SMD, upper CI, tau-squared and number of studies. Hedges’g SMD and 95% CI were calculated using Comprehensive Meta-Analysis (version 3.3; Biostat Inc., Englewood, NJ) based on sample size, means and pooled pre- and post-SDs for the within-group analyses and pooled post SDs for the between-group analyses. Variation of the true effect size was presented with 95% prediction intervals (PI), calculated based on SMD, upper CI, tau-squared and number of studies.

In addition, given that the analyses require pre-post correlation coefficients (r), we used the open dataset in one of the included studies Saeterbakken [4] and applied the calculated r in all analyses. Notably, maximal dynamic strength; *r* = 0.84, isometric strength; *r* = 0.79, countermovement jump; *r* = 0.87 and hypertrophy; *r* = 0.92. However, since the r was obtained only from one study, we also performed sensitivity analyses to determine if the results were robust with lower and more conservative correlation coefficients (*r* = 0.5 and 0.7) [42].

Maximal dynamic strength was compared in two strength-specific analyses: free-weight strength changes in both training groups and machine strength changes in both training groups. Since several of the included studies compared dynamic strength in more than one pair of exercises, we chose to compare the exercises that most studies compared: squat as a free-weight exercise (lunges in one study) with the machine equivalent multi-joint exercise (leg press, hack squat or smith machine squat). Other lower-body exercise comparisons were excluded in the strength-specific analyses equivalent exercise (leg press, hack squat, smith machine squat or one study with knee extensions). Other lower-body exercise comparisons were excluded [23, 32, 34]. Similarly, given most studies tested chest exercises, other upper-body exercises were excluded [23]. Thus, bench press as a free-weight upper-body exercise was compared with a machine equivalent exercise (Smith machine bench press, lying chest press or seated chest press). If a study had both lower- and upper-body comparison exercises, the two exercises were pooled into one SMD estimate (average and pooled standard deviation) to prevent unit-of-analysis-error [23, 38]. The lying chest press exercises were also included instead of the seated chest exercises in the two studies by Lennon [35] and Mayhew [36] to match regular lying free-weight bench press. However, seated chest press was included from the study by Langford [12] since no lying machine alternative was available. Additionally, the underweight- and the obese participants in Mayhew [36] were pooled into one SMD estimate within the free-weight and machine group comparisons, however this study was not included in the machine strength analysis since the free-weight group did not test strength in machines [36]. Whilst the study by Prieto-González and Sedlacek [24] was excluded from the strength analyses given they tested only strength in free-weight exercises that none of the groups trained.

The isometric strength analysis measured lower-body isometric strength as newton, whilst the countermovement jump analysis included changes in jump height (cm). Fry [34] did not report the isometric strength values, hence we read of the graphs using a web-based tool to extract numerical data from graph images whereby two endpoints on both the X and Y axis were chosen before entering the identical reference values from the figures, followed by manually selecting the mean and SD with the software (https://apps.automeris.io/wpd/). The summed isometric strength value of both legs was used in the study by Wirth [40]. Additionally, the most stable isometric and countermovement jump test conditions in Saeterbakken [4] was chosen for both groups over the unstable test conditions to be more comparable to the other studies. Since few studies assessed changes in muscle size, we included all measurement methods in the analysis assessing hypertrophy (whole-body fat-free mass, kg [Air Displacement Plethysmograph, BodPod], muscle thickness, mm [ultrasound], limb or chest circumferences, cm and estimation of fat and thereby fat-free mass from skinfold measurements, kg. The average muscle-rested-circumference measurement values for the upper arm, thigh and chest were used as the SMD for hypertrophy from Aerenhouts & D'hondt [23]. In addition, the two ultrasound measurements of biceps and quadriceps by Schwanbeck et al. were pooled into one SMD estimate.

Furthermore, to directly compare strength changes in the exercises that each group trained (free-weights or machines) we conducted an additional analysis (a direct effect size comparison). In other words, the effect size of strength changes in free-weight tests for the free-weight training groups were compared with the effect size of strength changes in machine tests for the machine-based training groups. Notably, the scale of free-weight exercises is not directly comparable to the scale of machine-based exercises; thus, a straightforward comparison of the pre-post changes would not be appropriate in the same SMD calculation. For example, squat strength could increase by 35 kg (pooled pre-post SD: 35) whilst leg press strength by 66 kg, (pooled pre-post SD: 61), both equating to a similar pre-post effect size of ~ 0.8, but a between group ES would be 0.5 in favour of machines [37]. Thus, the SMDs from the pre-post within group analysis was used as a standardized value and a subsequent single SMD estimate “synthetic effect-size” was calculated where SMDs machine were subtracted with SMDs free-weight and the variance from the two initial pre-post analysis were merged [43]. Meta-analysis was then performed on the synthetic effect-size. Equally, sub analyses were performed for the dynamic strength upper-body exercises, lower body exercises and training status with the same direct-strength-comparison approach. Significant levels for the differences between training status (strength-trained vs. untrained) and lower- vs. upper body were tested with the Q-test for heterogeneity between sub-analysis. In addition, a within group meta-analyses were conducted to test whether the free-weight- and machine training groups significantly increased from baseline in maximal dynamic- and isometric strength, countermovement jump height and muscle hypertrophy.

Sensitivity analyses were carried out to check if any individual study had a large impact on the results by removing one study at a time from the analyses. Assessment of heterogeneity and variance were checked (Cochran’s Q test, I^2^ and T^2^) in addition to publication bias by Egger’s regression test, Duval and Tweedie’s trim and fill method and visual inspection of the funnel plots for asymmetry by plotting standard errors against Hedges’ g values. An alpha level of p ≤ 0.05 was set as the criterion for statistical significance with the following SMD classifications of magnitude: small (≤ 0.2), medium (0.2–0.5), large (0.5–0.8), and very large (> 0.8) [44]. Results are reported as mean ± 95% CIs and the software Comprehensive Meta-Analysis (version 3.3; Biostat Inc., Englewood, NJ) was used to run the statistics.

## Results

**Fig. 1:**
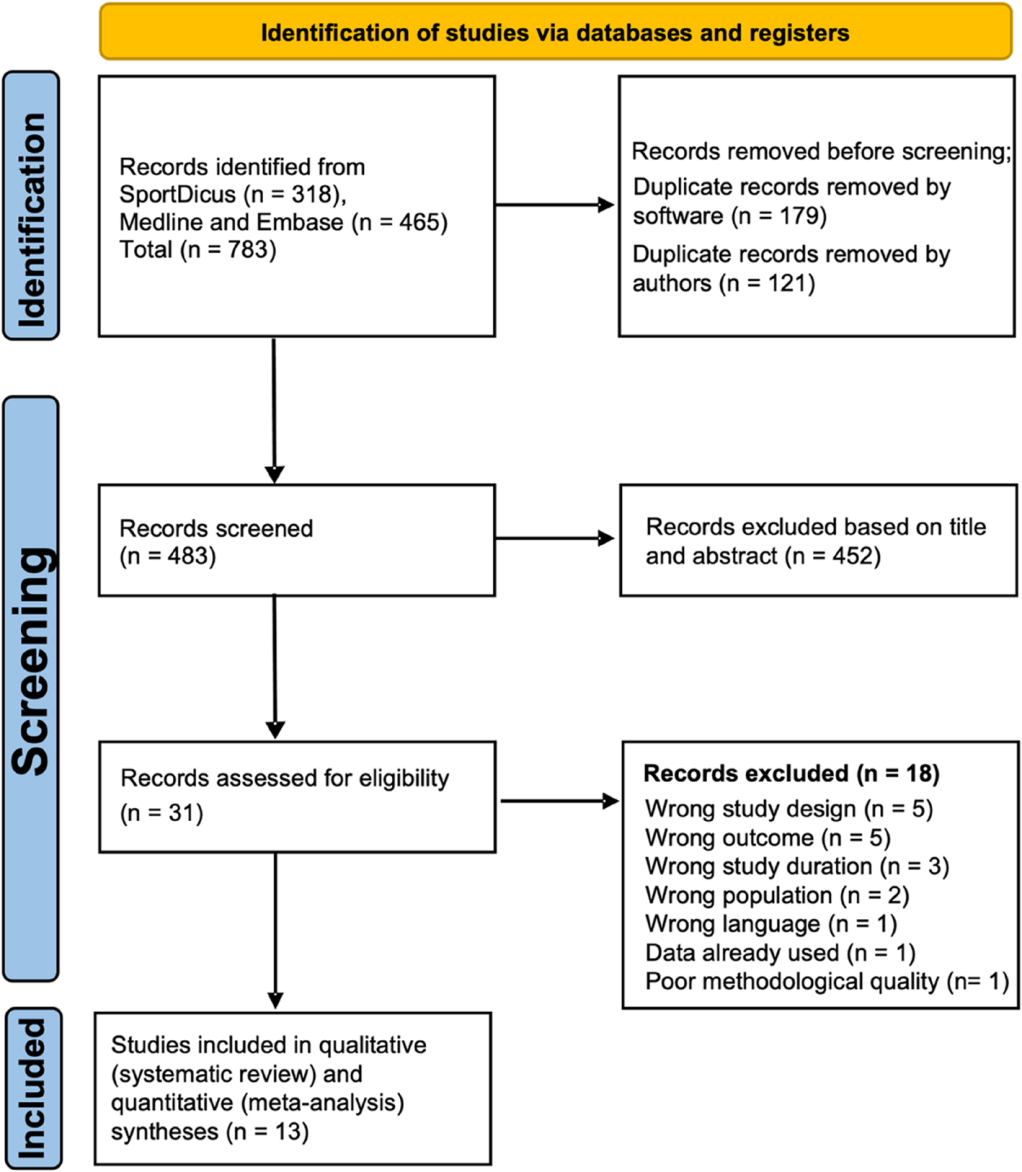
Flow chart of the PRISMA process showing the number of records collected, number of articles screened, number of articles excluded with reason, and the final number of articles included in the analysis

A total of 704 studies were evaluated based on the initial results of the search and reduced to 429 after duplicates were removed. Following the title and abstract screening, 30 studies were reviewed in full text. Subsequently, 13 studies that fulfilled the inclusion criteria were included [4, 12, 23, 24, 27, 32, 34–40]. Furthermore, the reference list of all included studies was checked for potential studies missing from the initial search, but no additional studies were observed. The search process is shown in Fig. 1.

### Characteristics of the included studies

Six of the studies involved trained [4, 12, 27, 32, 37, 40] and seven involved untrained participants [23, 24, 34–36, 38, 39]. None of the studies examined competitive athletes. The total numbers of participants in the studies were *n* = 1016, with 789 men and 219 women with an average sample size of 72.6 ± 111.7 (range 19–429). Two studies included both men and women [32, 38] and two studies included only women. The average duration of the studies was 8.9 ± 1.9 weeks (range 6–12), and the training frequency was from two to four sessions per week. Common exercises in the free-weight groups were barbell exercises such as the back squat and bench press, whereas leg press, chest press and various Smith machine exercises were often used in the machine groups. Participant’s characteristics along with other details form the included studies are listed in Table 2. Six studies assessed muscle hypertrophy [4, 23, 24, 36–38], whereby two measured muscle size by ultrasound [4, 38], two used body composition estimates from bod pod [37, 38], two used skinfolds to estimate body composition [24, 36], and one used circumferences [23]. The muscular strength assessment was obtained through strength tests with the following methods: 1 RM [23, 24, 35–37, 39, 40], 3 RM [12], 6 RM [27, 28] 6–10 RM [38], and 10 RM [4].

### Quality assessment

Table 3 presents the results of the quality assessment. The average score was 8.6. Five studies were rated as fair quality [12, 24, 34, 35, 40]. Eight studies were rated as good [4, 23, 27, 32, 36, 37, 39]. None of the studies was rated as having poor or excellent methodological quality. The raters average mean sum scores on the studies (*n* = 13) were 7.36 (SD = 1.15) and 7.64 (SD = 1.15), respectively. The intraclass correlation (two-way-mixed effect, absolute agreement) for the two raters was 0.85, with 95% Confidence Interval: 0.54 to 0.95, with *p* = 0.001.

**Table 3:** Quality assessment using the TESTEX checklist. 1 = criteria met; 0 = criteria not met

	**1**	**2**	**3**	**4**	**5**	**6a**	**6b**	**6c**	**7**	**8a**	**8b**	**9**	**10**	**11**	**12**	**Total score (max 15 p)**
**Aerenhouts & D’hondt 2020** [23]	1	1	0	1	0	0	1	0	0	1	1	1	0	1	1	**11**
**Augustsson et al., 1998** [32]	1	0	0	1	0	1	0	1	0	1	1	1	1	1	1	**10**
**Fry et al., 1992** [34]	0	0	0	1	0	0	0	0	0	1	1	1	1	1	1	**7**
**Langford et al., 2007** [12]	0	0	0	1	0	0	0	0	0	1	1	1	1	1	1	**7**
**Lennon et.,al 2010** [35]	0	0	0	1	0	1	0	0	0	1	0	1	0	1	1	**8**
**Mayhew et.,al 2010** [36]	1	0	0	1	0	1	0	0	0	1	1	1	0	1	1	**9**
**Prieto-Gonzalez &Sedlack 2021** [24]	0	0	0	1	0	0	0	0	0	1	1	1	1	1	1	**7**
**Rossi et al.,2018** [37]	1	0	1	1	0	1	0	0	0	1	1	1	0	1	1	**9**
**Saeterbakken et al.,2019** [4]	1	0	1	1	0	1	0	0	0	1	1	1	0	1	1	**9**
**Saeterbakken et al.,2016** [27]	1	1	0	1	0	0	0	0	0	1	1	1	1	1	1	**9**
**Schwanbeck et al.,2020** [38]	0	1	0	1	0	0	0	0	0	1	1	1	0	1	1	**9**
**Schwarz et al., 2019** [39]	1	0	0	1	0	1	0	0	0	1	1	1	0	1	1	**9**
**Wirth et al., 2016** [40]	0	0	0	1	0	1	0	0	0	1	1	1	0	1	1	**8**
**Sum**	**7**	**3**	**1**	**13**	**0**	**7**	**1**	**2**	**0**	**13**	**13**	**13**	**13**	**13**	**13**	

For the studies from Saeterbakken [27] and Augustsson [32], there were a few minor disagreements on the Testex quality assessment of items 1, 4 and 6. To reach 100% consensus on the quality assessment, disagreements were solved through discussion between the two raters. The final rating scores are listed in Table 3.

### ***The ***effect*** of free-weight versus machine-based training on maximal dynamic strength***

**Fig. 2:**
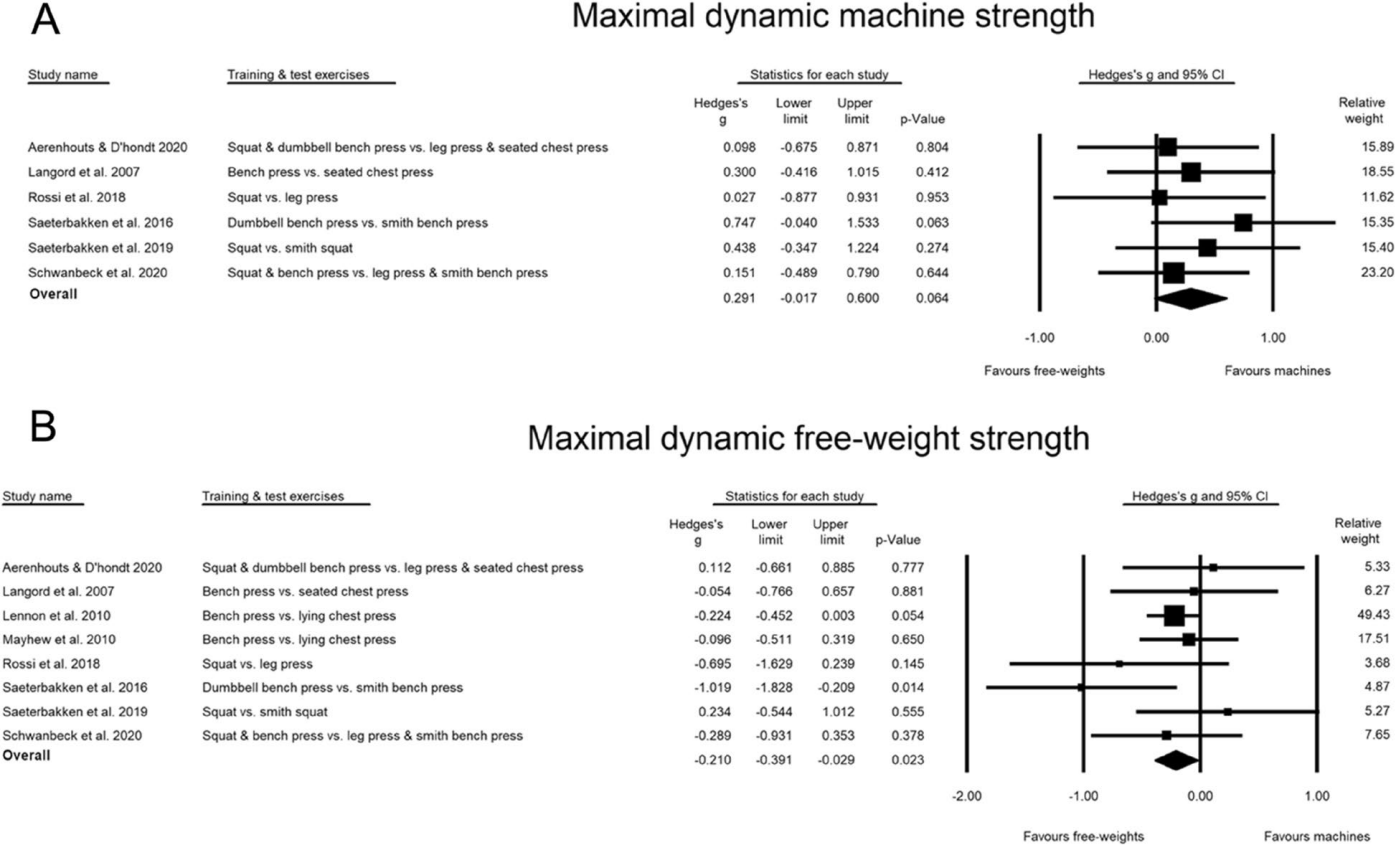
Forest-plot which shows the effect size changes when both groups tested machines (**A**), and when both groups tested free-weights (**B**)

The meta-analyses on changes in maximal strength in free-weight exercise tests observed a significant greater increase in the free-weight training group than in the machine training group (SMD: -0.210, CI: -0.391, -0.029, PI: -0.484, 0.064, *p* = 0.023) (Fig. 2A), with no difference between trained and untrained (SMD: -0.356 and -0.185, respectively (SMD: -0.356 and -0.185, respectively, *p* = 0.574). Maximal strength in machine-based strength tests tended to increase more in the machine group than the free-weight group (SMD: 0.291, CI: -0.017, 0.600, PI: -0.147, 0.729, *p* = 0.064) (Fig. 2B), with no difference between trained vs. untrained ((SMD: 0.385 and 0.129, respectively) SMD: 0.385 and 0.129, respectively, *p* = 0.427). The results were consistent across sensitivity analyses (with *r* = 0.5 and 0.7). However, removal of Lennon [35] from the free-weight exercise test analysis would have adjusted the point estimate from significant to non-significant (SMD: -0.207 [CI: -0.490, 0.077], *p* = 0.153), and removal of each of the two studies by Saeterbakken et al. [4, 28] from the machine test analysis adjusted the point estimate from a tendency to non-significant values (SMD: 0.209–0.265 [CI: -0.126–0.6], *p* = 0.122–0.222).

The meta-analyses on strength found maximal strength in free-weight exercise tests to increase significantly more in the free-weight group than in the machine group (SMD: -0.217, CI: -0.382, -0.053, PI: -0.415, -0.019, *p* = 0.010), with no difference between trained and untrained (*p* = 0.490). Maximal strength in machine-based strength tests tended to increase more in the machine group than the free-weight group (SMD: 0.243, CI: -0.046, 0.531, PI: -0.134, 0.621, *p* = 0.099), with no difference between trained vs. untrained (*p* = 0.578). The results were consistent across sensitivity analyses (with *r* = 0.5 and 0.7), however, removal of Lennon [35] from the free-weight exercise test analysis would have adjusted the point estimate from significant to a trend (SMD: -0.220 [CI: -0.477, 0.038], *p* = 0.094).

### The effect of free-weight training on free-weight strength versus machine-based training on machine-based strength (direct-strength-comparison-analysis)

**Fig. 3:**
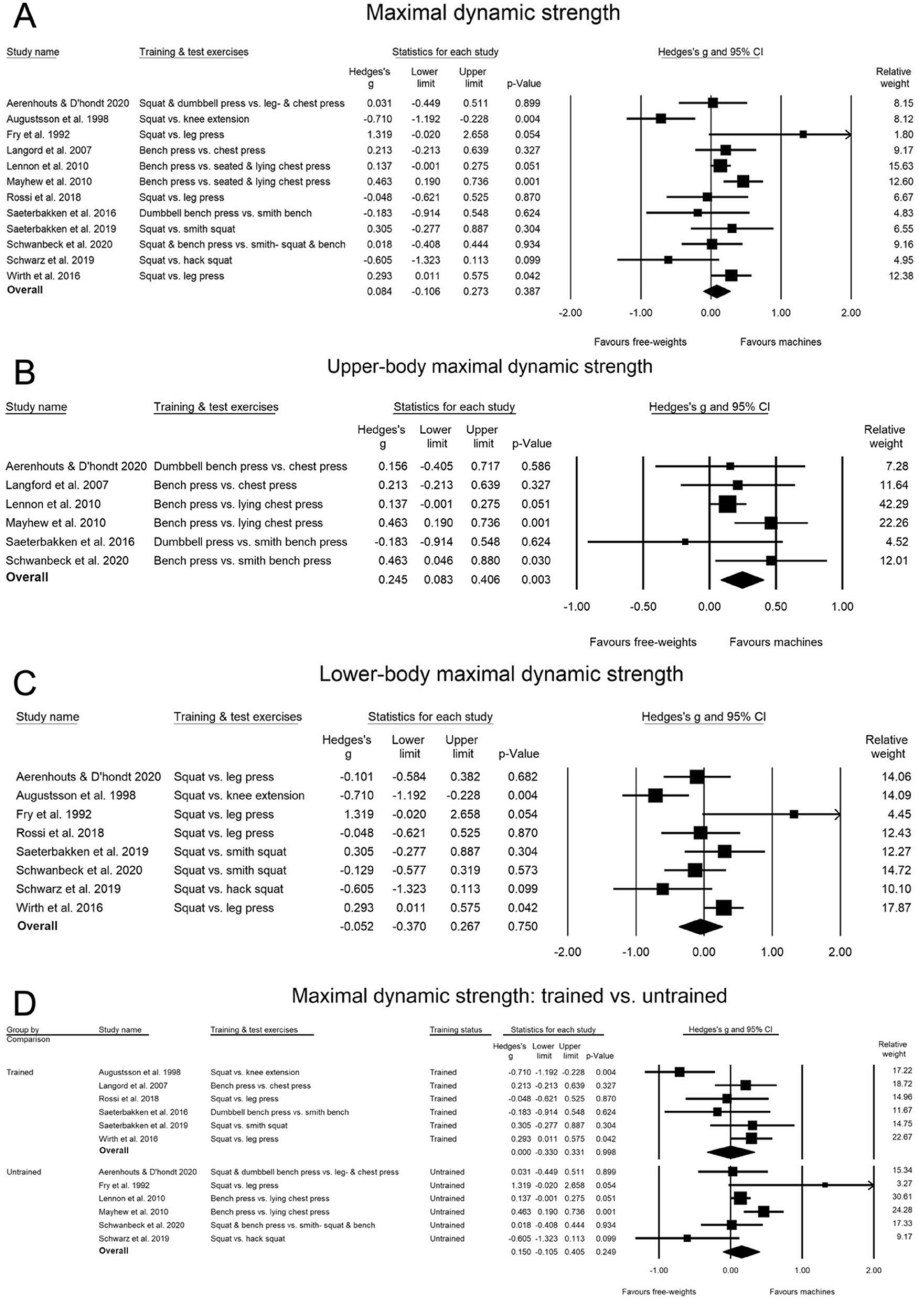
Forest-plot that shows the effect of free-weight training on changes in free-weight strength, versus machine-based training on the changes in machine-based strength (direct-strength-comparison-analysis), in maximal dynamic strength (**A**), maximal dynamic strength in the upper-body (**B**), maximal dynamic strength in the lower-body (**C**), and the influence of training status (**D**) This is the effect of free-weight training on free-weight strength versus machine-based training on machine-based strength (direct-strength-comparison-analysis)

In the direct-strength-comparison-analysis (machine group tested machine-based strength versus free-weight group tested in free-weight strength), no significant differences were observed between the free-weight groups versus the machine groups (machine ES—free-weight ES: 0.084 (CI: -0.106, 0.273, PI: -0.481, 0.649, *p* = 0.387)) (Fig. 3A). The results were similar with correlation coefficients of 0.7, whereas a trend was observed with correlation of 0.5 with greater strength increase in machine-based training (SMD 0.202, CI: -0.034, 0.438, *p* = 0.094). Furthermore, the sensitivity analysis found that removal of Augustsson [32] adjusted the results to significantly favour strength changes in the machine group (SMD: 0.187 [CI: 0.041, 0.334], *p* = 0.012). Training status did not influence the results (*p* = 0.483), strength-trained ES: 0.0005 (CI: -0.330, 0.331, *p* = 0.998) and untrained ES: 0.150 (CI: -0.105, 0.405, *p* = 0.249) (Fig. 3D). The direct-strength-sub-analyses revealed no differences between upper- vs. lower-body (*p* = 0.104); upper-body strength increased more in the machine group compared to the free-weight group (SMD: 0.245, CI: 0.083, 0.406, *p* = 0.003) (Fig. 3B), but no group-differences were observed in the lower-body strength analysis (SMD: -0.052, CI: -0.370, 0.267, *p* = 0.750) (Fig. 3C). The one-study removal sensitivity analyses did not change the results.

### The effect of free-weight- versus machine-based training on isometric strength

**Fig. 4:**
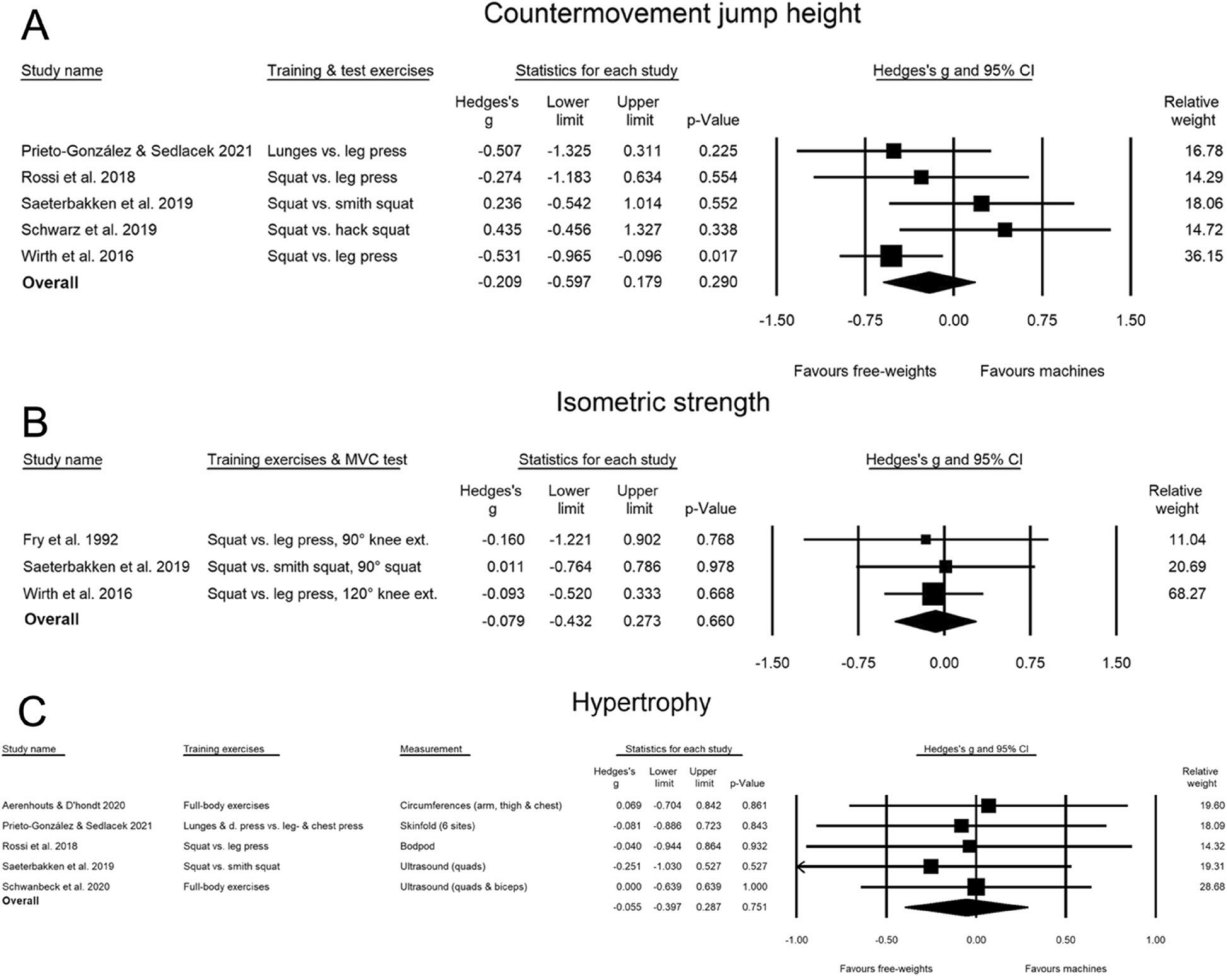
Forest-plot which shows the effect size changes in countermovement jump (**A**), isometric strength (**B**), and hypertrophy (**C**)

No significant difference was observed between the free-weight vs. machine group in isometric strength (SMD: -0.079 [CI: -0.432, 0.273, PI: -2.365, 2.207, *p* = 0.660) (Fig. 4B) and the sensitivity analyses did not influence the results.

### The effect of free-weight- versus machine-based training on countermovement jump height

No significant difference was observed between the free-weight vs. machine group in countermovement jump performance (SMD: -0.209 [CI: -0.597, 0.179, PI: -1.208, 0.790, *p* = 0.290) (Fig. 4A) and the results were robust across different correlation coefficients. However, removal of the study by Schwarz [39] from the analysis adjusted the point estimate from a non-significant effect to significantly favor the free-weight group (SMD: -0.364, [CI: -0.686, -0.042, *p* = 0.027).

### The effect of free-weight- versus machine-based training on hypertrophy

The meta-analysis revealed no significant differences in hypertrophy between the free-weight vs. the machine group with a SMD of -0.055 (CI: -0.397, 0.287, PI: -0.611, 0.500, *p* = 0.751) (Figs. 3 and 4C). Sensitivity analysis and correlation coefficients did not influence the result.

#### Heterogeneity and risk of bias for the between groups analyses

Cochran’s Q test for heterogeneity found no significant between-study variance: maximal dynamic free-weight strength: Q = 7.49, I^2^ & T^2^ = 0.00 *p* = 0.49; maximal dynamic machine strength: Q = 2.91, I^2^ & T^2^ = 0.00 *p* = 0.82; maximal dynamic strength analysis: Q = 27.7, I^2^ = 60.28 & T^2^ = 0.06, *p* = 0.39; isometric strength analysis: Q = 0.08, I^2^ & T^2^ = 0.00, *p* = 0.96; isometric strength analysis: Q = 0.08, I^2^ & T^2^ = 0.00, *p* = 0.66; countermovement jump analysis: Q = 5.7, I^2^ = 30.25, T^2^ = 0.06, *p* = 0.29 and hypertrophy analysis: Q = 0.38, I^2^ & T^2^ = 0.00, *p* = 0.98. However, heterogeneity was significant in the direct-strength-comparison-analysis: Q = 27.68, I^2^ = 60.25 & T^2^ = 0.05, *p* = 0.004. Visual inspection indicated three studies outside the publication bias plot in the direct-strength-comparison-analysis (two to the left and one to the right of the plot) but none in the other analyses. Moreover, the Duval and Tweedie’s trim and fill analysis method observed one missing study to the right of the funnel plot in the maximal dynamic free-weight strength analysis, but it did not significantly influence the results. Additionally, one study to the left of the plot was missing in the countermovement jump analysis, which also did not significantly impact the results. Furthermore, Eggers’s test for funnel plot asymmetry did not indicate potential publication bias in any of the analyses (*p* = 0.22–0.81).

### Pre- post within group changes in maximal dynamic- and isometric strength, countermovement jump and hypertrophy (direct effect size comparison)

**Fig. 5:**
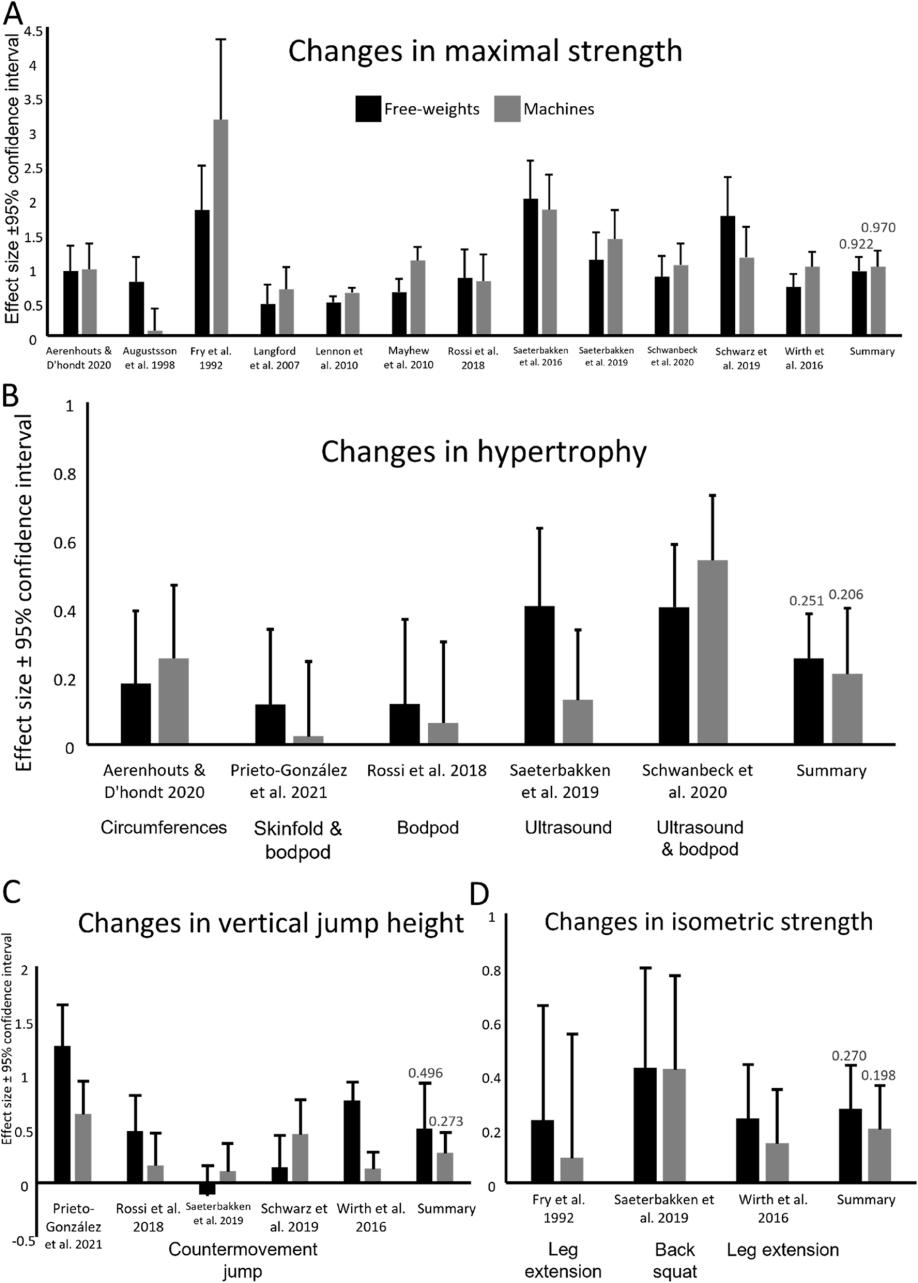
Shows the effect sizes (mean ± SD) of changes from pre-test to post-test in maximal strength (**A**), hypertrophy (**B**), vertical jump height (**C**) and isometric strength (**D**). Black are changes in free-weight when participants trained with free-weights and grey are changes in machine groups when participants trained with machines

Maximal dynamic free-weight strength increased from pre to post test in the free-weights group with an SMD of 0.922 (95% CI: 0.713, 1.131, PI: 0.169, 1.675, *p* < 0.0001), whereas the machines group increased strength in the machine exercises with an SMD of 0.970 (CI: 0.738, 1.202, PI: 0.124, 1.815, *p* < 0.0001), shown in fig. 5A. Isometric strength increased from pre to post test in the free-weight group (SMD: 0.270, CI: 0.110, 0.431, PI: -0.769, 1.310, *p* = 0.001), and in the machine group (SMD: 0.198, CI: 0.036, 0.361, PI: -0.863, 1.260, *p* = 0.017), shown in fig. 5D. Countermovement jump height increased from pre to post training in the free-weight group (SMD: 0.496, CI: 0.067, 0.925, PI: -1.140, 2.132, *p* = 0.024), and in the machine group (SMD: 0.273, CI: 0.082, 0.465, PI: -0.354, 0.901, *p* = 0.005), presented in fig. 5C. Similarly, hypertrophy increased from pre to post test in the free-weight group (SMD: 0.251, CI: 0.121, 0.381, PI: -0.129, 0.631, *p* < 0.0001) and in the machine group (SMD: 0.206, CI: 0.015, 0.398, PI: -0.473, 0.886, *p* = 0.035), presented in fig. 5B.

## Discussion

The purpose of the present study was to systematically review and summarize the data on the effect of free-weight vs. machine-based strength training on maximal strength, hypertrophy and countermovement jump performance. Our main findings were that when both groups tested maximal strength with free-weights, free-weight training increased strength more, and a tendency was observed towards a larger increase in maximal strength in machines with machine-based strength training. However, when effect sizes from each respective training and test modality were compared (free-weight strength change effect size after free-weight training, versus machine strength change effect size after machine-based training), there was no difference between free-weight and machine-based strength training for increases maximal dynamic strength (ES free-weight group: 0.922, ES machine group: 0.970), isometric strength (ES free-weight group: 0.0.270, ES machine group: 0.198), hypertrophy (ES free-weight group: 0.251, ES machine group: 0.206) and countermovement jump performance (ES free-weight group: 0.496, ES machine group: 0.273). The sub-analysis of the direct effect size comparison revealed a significant difference between training modalities towards larger increases with machine-based training for upper-body strength, but no difference for lower-body strength. Further analysis revealed that both modalities lead to a significant pre-post increase in maximal strength, countermovement jump heigh, isometric strength and hypertrophy. For training status, we found no difference between modalities for neither free-weights nor machines.

### Maximal strength

The free-weight group increased strength more when both groups were tested in free-weight exercises, and the machine group tended to increase strength in machine exercises more than the free-weight group. This follows the “specific adaptations to imposed demands” (SAID) principle [45]. A possible explanation to why the specific strength analyses only revealed a statistically significant difference when both groups tested strength with free-weight exercises could be the complexity of the exercises. Free-weights involve more degrees of freedom compared to machines [46], therefore the transfer from free-weights to machine can be classified as a transition from the most complex task to the least complex task, which could potentially be an easier transfer compared to the other way around. However, sensitivity analysis revealed that the removal of Augustsson [32] resulted in an advantage for machine-based strength training in the direct effect size comparison. This could imply that less degrees of freedom could yield the best results, but a study by Rutherford & Jones found the largest relative increase in leg extension for the group who had the most degrees of freedom [47]. A possible explanation for this could be the learning effect, thus studies with longer duration or familiarization could answer this more clearly. The specificity of the strength adaptations suggests that recreational athletes who only want to get stronger can base their choice on personal preference as free-weights and machines resulted in the same relatively increase in strength. However, athletes in sports like powerlifting, weightlifting and other sports with a specific exercise requirement should prioritize those exercises in their training.

We did not observe any differences in maximal strength changes between free-weight and machine-based strength training when assessed with exercises like their respective training interventions. But sensitivity analysis revealed that removal of the Augustsson et al. [32] adjusted the results in favour of machine-based strength training. The machine group in that study saw a small progression (2.2%) compared both to the free-weight (30.8%), but also compared to the machine group in other studies (15.8–42.2%). The small changes in strength after machine-based training in Augustsson et al. [32] can possibly be explained by the equipment used. They trained with isoinertial exercises but were the only study that measured strength in an isokinetic knee extensor machine and who used single-joint movement in the machine-based group. The isokinetic knee extensor differs from a normal knee extensor and normal machines due to a movement with constant speed and removal of the stretch–shortening cycle. The transfer from isointertial machine-based training to measurements of maximal strength in isokinetic testing [32], and the relative short duration of the intervention (6 weeks) [32], could have hampered effect size of machine-based strength changes in Augustsson et al. [32]. In contrast, the differences between modalities could also be due to different requirements for stability. In line with increased demands for stability, the brain starts to focus more on stability at the expense of force production [18, 48].

The direct-strength-sub-analyses revealed a difference between free-weights and machine-based strength training for upper-body in favour of machines, whilst no differences were detected for the lower body. A possible explanation for the lack of difference in the lower body could be the transfer of daily life to the squat exercise. The squats are quite like many of our daily activity as standing up and sitting down. This echoes the Woodworth & Thorndike [49] identical elementers theory which builds on similarity in proccecing and execution.

We performed a sub-analysis for training status with the direct-strength data. There was an even split between trained [4, 12, 27, 32, 37, 40] and untrained athletes [23, 34–36, 38, 39]. We found no difference between modalities in trained or untrained. However, sensitivity-analysis revealed a difference towards machines in trained participants Augustsson [32], and removal of Schwarz [39] revealed a difference towards machines in untrained participants, and only one study [39] leaned towards free-weights. This indicates that for unexperienced lifters, machines could potentially induce lager larger strength changes due to less stability requirements and degrees of freedom [46]. This could help trainers to focus on force production with less requirements for stability. Moreover, based on the possible higher risk of injury at free-weights [1, 8, 25], a recommendation to use machines for unexperienced could be made. Experienced trained lifters that have more developed techniques could likely diminish the potential increase difficulty through stability and degrees of freedom [46]. Based on this we recommend experienced lifters to choose modality based on training goal (i.e., specificity) and personal preference.

No differences between free-weight and machine-based strength training were detected in the three studies with isometric strength measurements. These results suggest that transfer of dynamics strength to isometric strength is similar between free-weight and machine-based strength training. Changes in countermovement jump performance did also not differ significantly between groups, but the results leaned towards the free-weight group. In support of this, sensitivity analysis revealed that the removal of Schwarz [39] skewed the results in favour of free-weight group. Four out of the five studies used the barbell squat in the free-weight group. It is important to note that the sample size was small, and one should be careful with the interpretation and generalisation into larger scale. The execution of the back squat is more like the execution of countermovement jumps than leg press, smith machine squat and hack squat. Hence, these results could be explained by specificity in the degrees of freedom [46], the direction force and the movement pattern. On the other hand, the leg press is often positioned lying supinated in 45 degrees. This reduces the hip extension contribution in the last part [50]. This reduces the transfer from one movement to another due to similarity in the movement/execution and similarity in cognitive processing [49]. Thus, athletes and others that needs to improve CMJ performance can potentially achieve better results with free-weight training due to the similarity in execution.

### Hypertrophy

Our analysis found no difference between free-weight and machine-based strength training for hypertrophy. Both training groups induced similar hypertrophic gains during the strength training interventions lasting nine weeks on average (ES free-weight group: 0.251, ES machine group: 0.206).

The included studies used varying methods for the estimation of muscle mass (muscle thickness with ultrasonography [4], limb and chest circumference [23], skinfold and bod pod measurements for estimation of body composition and fat-free mass [24], bod pod [37] and ultrasonography in combination with bod pod [38]). These methods vary greatly in their ability to detect changes over time, and the uncertainty around circumference and skinfolds for muscle mass estimation highlights the need for more studies with direct hypertrophy measurement. Due to the diversity in the measurements of muscle mass and the sparse number of included studies, one should be careful with the interpretation of the results.

Based on studies showing more myoelectrical activity in synergist muscles with free-weight exercises than machine-based strength training [6, 14, 51], one could speculate that free-weights could lead to more total muscle growth due to an increased activation of the synergists. This meta-analysis could not confirm those speculations, with equal gains in fat-free mass of the five studies that included measurement of body composition [4, 23, 24, 37, 38]. In contrast to the speculations of gains in total muscle mass, the degree of failure in the main muscle groups with free-weight exercises could be limited by failure in smaller synergist muscles and a failure to conduct the exercises with proper techniques. Therefore, it could be speculated that machines can train the main muscle group(s) closer to failure and thus induce more gains in the main muscles of the exercise. Importantly, muscle growth seems to be similar even with large variations in load (> 30% of 1RM) if it is performed in proximity to failure [52]. For example, Mitchell and colleagues [53] found that 30% and 80% of one-repetition maximum (1 RM) knee extensions elicit similar muscle growth. Training close enough to failure in the execution of the exercise is possible with both modalities, but machine-based strength training could potentially isolate specific muscle groups more easily than free-weight training. For example, trunk musculature could potentially limit the performance in a front squat and in a standing barbell shoulder press. This could lead to other muscles than the target muscles (i.e., quadriceps, glutes, and anterior deltoid) to be the limiting factor.

Machine’s ability to isolate a certain muscle or group could be desirable for bodybuilder or athletes that aim to improve muscle size and strength in specific muscle groups, or specific joint or muscle rehabilitation after injury. Our results correspond with the previous meta-analysis by Heidel [5]. The aforementioned meta-analysis included three studies [4, 37, 38], but we included two more studies [23, 24]. None of those studies changed the current perspective on free-weights versus machine-based strength training on the development of muscle hypertrophy.

Summed up, total muscle hypertrophy could be equal but differ in magnitude of regional muscle growth with free-weight and machine-based exercises. An example of such responses is the study from Costa [54], which found same total muscle growth between a group who performed the same exercises on every workout and a group who varied. But the regional growth was different. The group who varied exercises experienced a significant increase in all sites, i.e., proximal, middle, and distal sections of the muscle. The group who did not vary exercises experienced a significant increase in most, but not all parts of the muscle. That could indicate that different part of the muscles responds to different exercises. In addition, differing regional hypertrophy have been observed with free-weights vs. machine-based strength training [55–58]. Different types of knee extension have shown different type of muscle growth in the quadriceps. For example, the barbell back squat does not seem to induce hypertrophy of the rectus femoris muscle [55–57]. A probable explanation is that the rectus femoris crosses both the knee- and hip joint, and thus does not contribute much during simultaneous knee- and hip extension [59]. In addition, it does not get stretched under tension, which is considered an important stimulus for muscle growth [60–62]. In contrast, leg extension seems to induce robust hypertrophy in the rectus femoris [58, 63]. Probably because the muscle length is not altered over the hip. A study by Zabaleta-Korta et al. [63] found favourable muscle growth in all three parts of the rectus femoris for the leg extension group. On the other hand, Earp [56] found no significant increase in neither part of the rectus femoris when participants trained with the barbell back squat. Moreover, Earp [56] saw a significant increase in all parts of the vastus lateralis muscle whereas Zabaleta-Korta [63] did not reach a significant increase in the vastus lateralis for the leg extension group. Based on these studies, a difference in local hypertrophy could occur between free-weight and machines, both due to the degree of stability in the exercise modality, but also a variety of factors affecting muscle growth stimuli in exercise selections such as the specific biomechanics of the exercise, available ROM and resistance curve. Nevertheless, a direct comparison of regional muscle growth should be conducted to investigate potential discrepancies in free-weight and machine-based training per se. Based on the current body of evidence, one could speculate that the combination of free-weights and machine would be the optimal choice to maximize total muscle growth.

## Strength

A strength to this meta-analysis is the comprehensive direct comparison between the modalities. Moreover, we have included tests where they measure the trained modalities and when they test neutral or the opposite. I.e strength test where the free-weight group are testing either free-weight, isometric or machine-based training. We also calculated the agreement score between the authors who ranked the included studies with testex. Rayyan were used to perform blinded inclusion.

## Weaknesses

Still a lack of studies in this area, especially within the realm of muscle hypertrophy. The measurements used are different and not always gold standard. It is not adjusted for other, maybe more important factors such as range of motion, intensity, volume, and frequency.

## Conclusion

The result of this review found no differences in free-weight or machine-based strength training induced changes when measured in the same exercise modality as they are training, nor in a neutral test (isometric strength test). Furthermore, training status did not seem to influence strength changes between the two training modalities. When the goal is to maximise muscle hypertrophy individual preferences should dictate the choice, but we speculate that a combination could yield the best benefit. Due to limited number of studies, one should be careful with the interpretations of the results, especially for muscle hypertrophy.

## Practical Implications

The findings in this meta-analysis gives valuable information for strength, sport and physique coaches as well as athletes. There is advantages and disadvantages with both modalities. This article is another piece to the puzzle regarding designing training programs. This paper in combination with summary on range of motion [60–62], resting intervals [64, 65], exercise order [31], volume [66], intensity as proximity to failure [67] and different loading schemes [22, 68] and frequency [69], could provide great insight to the literature as a whole. However, one should always pay attention to the individual respond when considering the overall evidence. Lastly, the current evidence is tentative and could change as we gather more studies and knowledge in the future.

### Supplementary Information

**Additional file 1:** Appendix: systematic search strategy.

## Data Availability

On reasonable request data can be shared.
