# Supplementary material for: Effect of free-weight vs. machine-based strength training on maximal strength, hypertrophy and jump performance – a systematic review and meta-analysis
Source: BMC Sports Sci Med Rehabil. 2023 Aug 15;15:103. doi: 10.1186/s13102-023-00713-4 (PMC10426227; doi:10.1186/s13102-023-00713-4)
Supplement: Supplementary file 1 — Additional file 1: Appendix: systematic search strategy. [file 13102_2023_713_MOESM1_ESM.docx]

Appendix: systematic search strategy

SPORTDiscus via EBSCOhost

Date: until 31^th^ Dec 2022.

Top of Form

| **#** | **Query** | **Limiters/Expanders** | **Results** |
| --- | --- | --- | --- |
| S9 | S4 and S6 and S7 | Search modes - Boolean/Phrase | 318 |
| S8 | S4 and S7 | Search modes - Boolean/Phrase | 702 |
| S7 | S2 or S5 | Search modes - Boolean/Phrase | 308,703 |
| S6 | S1 or S3 | Search modes - Boolean/Phrase | 25,064 |
| S5 | power* or CMJ* or jump* or watt* or speed* or velocity* or maximal* or strength* or MVC* or rm* or force* or isometric* or "voluntary contraction*" or "repetition maximum*" or isokinetic* or torque* or hypertrophy* or "cross-sectional area*" or CSA* or "fat free mass*" or FFM* or "lean body mass*" or LBM* or "lean mass*" or (muscle* W0 (size* or thickness* or volume* or mass*)) | Search modes - Boolean/Phrase | 308,537 |
| S4 | (Free weight* or barbell* or dumbbell*) and (machine* or smith* or isokinetic*) or "stabil* training*" or "instabil* training*") | Search modes - Boolean/Phrase | 1,102 |
| S3 | (resistance* or strength*) W0 (exercise* or training*) | Search modes - Boolean/Phrase | 25,064 |
| S2 | (MH "Muscle, Skeletal") OR (MH "Muscle Strength") OR DE "MUSCLE strength" OR (MH "Hypertrophy") OR (MH "Adaptation, Physiological") OR (MH "Muscle Development") OR (DE "MUSCLE growth") OR (DE "MUSCULAR hypertrophy") | Search modes - Boolean/Phrase | 19,788 |
| S1 | (MH "Resistance Training") OR DE "RESISTANCE training" OR DE "STRENGTH training" | Search modes - Boolean/Phrase | 8,609 |

Bottom of Form

MEDLINE & Embase via Ovid

Date: until 31^th^ Dec 2022.

(Embase <1974 to 2022 December 31>, Ovid MEDLINE(R) ALL <1946 to December 31, 2022>)

| 1 | resistance training/ | 37498 |
| --- | --- | --- |
| 2 | ((resistance* or strength*) adj (training* or exercise*)).mp. [mp=ti, ab, hw, tn, ot, dm, mf, dv, kf, fx, dq, nm, ox, px, rx, ui, sy] | 64545 |
| 3 | skeletal muscle/ or muscle strength/ or hypertrophy/ or muscle development/ or adaptation/ or adaptation, physiological/ | 629462 |
| 4 | (power* or CMJ* or jump* or watt* or speed* or velocity* or maximal* or strength* or MVC* or rm* or force* or isometric* or "voluntary contraction*" or "repetition maximum*" or isokinetic* or torque* or hypertrophy* or "cross-sectional area*" or CSA* or "fat free mass*" or FFM* or "lean body mass*" or LBM* or "lean mass*" or (muscle* adj (size* or thickness* or volume* or mass*))).mp. [mp=ti, ab, hw, tn, ot, dm, mf, dv, kf, fx, dq, nm, ox, px, rx, ui, sy] | 5637986 |
| 5 | (((Free weight* or barbell* or dumbbell*) and (machine* or smith* or isokinetic*)) or ("stabil* training*" or "instabil* training*")).mp. [mp=ti, ab, hw, tn, ot, dm, mf, dv, kf, fx, dq, nm, ox, px, rx, ui, sy] | 1450 |
| 6 | 1 or 2 | 64545 |
| 7 | 3 or 4 | 5984448 |
| 8 | 5 and 6 and 7 | 465 |
| 9 | remove duplicates from 8 | 286 |
